# High glucose represses the proliferation of tendon fibroblasts by inhibiting autophagy activation in tendon injury

**DOI:** 10.1042/BSR20210640

**Published:** 2022-03-16

**Authors:** Fu-Chen Song, Jia-Qin Yuan, Mei-Dong Zhu, Qi Li, Sheng-Hua Liu, Lei Zhang, Cheng Zhao

**Affiliations:** 1 Department of Vascular Surgery, Yueyang Hospital of Integrated Traditional Chinese and Western Medicine, Shanghai University of Traditional Chinese Medicine, Shanghai, P.R. China; 2 Yueyang Clinical Medical College, Shanghai University of Traditional Chinese Medicine, Shanghai, P.R. China; 3 Department of Vascular Disease, Shanghai TCM-Integrated Hospital, Shanghai University of Traditional Chinese Medicine, Shanghai, P.R. China

**Keywords:** apoptosis, autophagy, Diabetic foot ulcer, Hyperglycemia, tendon fibroblasts

## Abstract

Diabetic foot ulcer (DFU) is a kind of common and disabling complication of Diabetes Mellitus (DM). Emerging studies have demonstrated that tendon fibroblasts play a crucial role in remodeling phase of wound healing. However, little is known about the mechanism underlying high glucose (HG)-induced decrease in tendon fibroblasts viability. In the present study, the rat models of DFU were established, and collagen deposition, autophagy activation and cell apoptosis in tendon tissues were assessed using Hematoxylin–Eosin (HE) staining, immunohistochemistry (IHC), and TdT-mediated dUTP Nick-End Labeling (TUNEL) assay, respectively. Tendon fibroblasts were isolated from Achilles tendon of the both limbs, and the effect of HG on autophagy activation in tendon fibroblasts was assessed using Western blot analysis, Cell Counting Kit-8 (CCK-8) assay, and flow cytometry. We found that cell apoptosis was increased significantly and autophagy activation was decreased in foot tendon tissues of DFU rats compared with normal tissues. The role of HG in regulating tendon fibroblasts viability was then investigated *in vitro*, and data showed that HG repressed cell viability and increased cell apoptosis. Furthermore, HG treatment reduced LC3-II expression and increased p62 expression, indicating that HG repressed autophagy activation of tendon fibroblasts. The autophagy activator rapamycin reversed the effect. More importantly, rapamycin alleviated the suppressive role of HG in tendon fibroblasts viability. Taken together, our data demonstrate that HG represses tendon fibroblasts proliferation by inhibiting autophagy activation in tendon injury.

## Introduction

Diabetes mellitus (DM) affects approximately 350 million people globally and its prevalence is rapidly increasing in recent years [1,2]. Diabetic foot ulcer (DFU) is one of the serious chronic complications in DM [3]. DFU commonly originates from poor glycemic control, infection of lower extremity vascular lesions, neuropathy, inadequate foot care or peripheral vascular disease [4,5], and generally leads to amputation of lower extremities and osteomyelitis of the foot.

Tendons could dynamically modulate their ability to store and deliver energy [6,7]. In tendon tissues, tenocytes, defined as Scleraxis (SCX)-expressed fibroblasts, comprise the bulk of cell population [6,8]. Tendon fibroblasts (or Tenocytes) exert a crucial physiological role in regulating tendon adaption, response to mechanical loading, and tendon repair after tissue injury [6,9]. Zeng et al., demonstrated that endothelial cells (ECs)-derived small extracellular vesicles (ECs-sEVs) activate skin fibroblasts autophagy, repress collagen synthesis, and result in a subsequent delay in the wound healing process [10]. Currently, the role of tendon fibroblasts in the progression of DFU remains unclear.

Autophagy is a highly conserved process that degrades misfolded proteins and damaged organelles to maintain intracellular homeostasis [11]. Therefore, autophagy acts as a protective mechanism in maintaining cell function and viability under physiological conditions. Emerging studies have demonstrated that autophagy exerts an essential role in β-cell health, and impaired autophagy is a factor in promoting β-cell dysfunction and diabetes progression [12,13]. On the contrary, autophagy is also considered as the second type of programmed cell death in which the accumulation of autophagosomes distinguishes itself from apoptosis [14]. Although current studies have confirmed that autophagy is involved in a variety of physiological and pathological processes, the role of autophagy in wound healing, especially in DFU, and the underlying mechanism was poorly understood.

Based on the above findings, here we investigated whether autophagy was activated or repressed in foot tendon tissues of DFU rats. Then the role of high glucose (HG) in regulating autophagy activation of tendon fibroblasts was assessed *in vitro*. The current results showed that cell apoptosis was increased, concurrently autophagy activation was decreased in tendons of diabetic foot patients compared with control. *In vitro* experiments, HG treatment promoted cell apoptosis and repressed autophagy activation in tendon fibroblasts by activating mTOR signaling.

## Materials and methods

### DM model

Male Sprague–Dawley rats (4–6 weeks old, 180–200 g) were purchased from Beijing Vital River Laboratory Animal Technology Co., Ltd. (Beijing, Shanghai). All procedures were approved by the Animal Ethics Committee of the Yueyang Hospital of Integrated Traditional Chinese and Western Medicine, Shanghai University of Traditional Chinese Medicine (IACUC number: YYLAC-2019-1). The rat model with DFU was established in experimental animal center of Shanghai University of Traditional Chinese Medicine as previously described [15]. In brief, after adaptive feeding for 7 days, rats were fed with high-fat and high-sugar diets for 4 weeks, followed by injecting intraperitoneally with streptozoticin (30 mg/kg body weight, Sigma–Aldrich, MO, U.S.A.). Streptozoticin was dissolved in sodium citrate solution. Control rats were fed with ordinary diets. Blood glucose levels were assessed using a blood gas analyzer after 3 days, and rats with the indicated blood glucose level (≥16.7 mmol/l, continuously for 10 days) were used for the rat model with DFU.

### Isolation of primary tendon fibroblasts

Rats were killed by cervical dislocation under anesthesia through intraperitoneal injection of pentobarbital (40 mg/kg). The Achilles tendon of the both limbs were isolated immediately in accordance with the aseptic operation as previously reported [16]. In brief, tendons were incubated with Ringer solution supplemented with 0.3% collagenase (Sigma–Aldrich) at 37°C for 3 h. Tendon fibroblasts were dispersed and maintained in DMEM contained with 10% FBS, 0.001% penicillin-G and, 0.001% streptomycin (Sangon Biotech, Shanghai, China).

### Hematoxylin–Eosin staining

The foot tendon tissues of DFU rats were fixed with 4% formalin for 12–24 h and sectioned at 4 μm after conventional paraffin embedding. The sections were placed in xylene, followed by high to low concentration of alcohol, and finally placed in distilled water for 5 min. After staining with Hematoxylin solution for 5 min, the sections were rinsed with 1% hydrochloric acid ethanol for 30 s and placed into 0.5% Eosin solution to stain for 5 min. Finally, all sections were dehydrated in increasing concentrations of ethanol and xylene and neutral gum were used to seal the film and observed under an optical microscope.

### Immunohistochemistry

All the foot tendon tissue sections were fixed in 4% formalin overnight and embedded in paraffin with standard techniques. After washing with PBS, the sections were incubated with primary antibodies overnight at 4°C as following: anti-LC3 antibody (1:200, ab48394, Abcam) and anti-Collagen 1 antibody (1:200, ab254113, Abcam). After washing three times in PBS, the sections were incubated with goat anti-rabbit HRP-conjugated secondary antibody (1:5000; ab205718, Abcam) for 30 min at 37°C and stained with DAB and Hematoxylin. The stained slides were recorded using a Nikon D300 microscope (Nikon, Tokyo, Japan).

### TdT-mediated dUTP nick-end labeling assay

Cell apoptosis in foot tendon tissues was analyzed using a TdT-mediated dUTP nick-end labeling (TUNEL) assay kit (Roche, Basel, Switzerland) as per the manufacturer’s instructions. In brief, paraffin sections were prepared, dewaxed, and hydrated in graded ethanol (70, 95, 100%), and then incubated with fluorescein isothiocyanate (FITC)-labeled dUTP and terminal deoxynucleotide transferase for 1 h at 37°C. Nuclei counterstaining was performed using DAPI (Sigma–Aldrich). Stained cells were observed under a fluorescence microscope and the percentage of TUNEL-positive cells was determined.

### Cell viability assay

Cell viability was measured using a cell counting kit-8 (CCK-8) assay according to the manufacturer’s instructions. Tendon fibroblasts were seeded in 96-wells plates (4 × 10^3^ cells/well) and treated with HG (25 mM) or rapamycin (100 nM; Rapa; Sigma–Aldrich) for different times (0, 24, 48 or 72 h), CCK-8 solution (10 μl, Dojindo Japan) was added and the cells were incubated for 2 h at 37°C. Absorbance was measured at a wavelength of 450 nm using a microplate reader (Thermo Scientific, Waltham, MA, U.S.A.). The assays were repeated three times.

### Cell apoptosis analysis

Annexin V-FITC/PI Apoptosis Detection Kit (Becton Dickinson Biosciences, San Jose, CA, U.S.A.) was used to determine the cell apoptosis as the manufacturer’s protocol. Briefly, the tendon fibroblasts (5 × 10^5^ cells) were treated with HG (25 mM) or rapamycin (100 nM) for 72 h and collected, washed with cold PBS, and resuspended with 400 μl binding buffer. Half of the cells were added with 5 µl Annexin V-FITC and incubated away from light for 15 min at room temperature. Then, 10 µl PI (Becton Dickinson Biosciences) was added into cells and the control (the rest half of cells). A BD FAC Scan Flow Cytometer (BD, Mountain View, U.S.A.) was used to assess and calculate the percentage of apoptotic cells.

### Western blot analysis

The tendon fibroblasts were harvested and the protein was extracted with RIPA lysis buffer (Beyotime, Shanghai, China). The protein concentration was calculated using a bicinchoninic acid protein assay kit (Beyotime). Equivalent amounts of proteins (35 μg) from each sample were separated by 10% SDS/PAGE gels and then transferred to polyvinylidene fluoride (PVDF) membranes (Merck Millipore, Billerica, MA, U.S.A.). The membranes were blocked in 5% non-fat dried milk for 1 h at room temperature and then incubated overnight at 4°C with the following primary antibodies: anti-LC3B (2 µg/ml, ab48394, Abcam), anti-P62 (2 µg/ml, ab91526, Abcam), anti-mTOR (1:2000, ab134903, Abcam), and anti-mTOR (phospho) antibody (1:1000, ab137133, Abcam). After washing with TBST, the membranes were incubated with Goat Anti-Rabbit H&L (HRP)-conjugated secondary antibodies (1:5000, ab205718, Abcam) at room temperature for 1 h. An ECL chemiluminescence kit (Millipore) was used to visualize the specific blots and autoradiograms were quantified by densitometry.

### Statistical analysis

Data were presented as the mean ± standard deviation (SD) of three independent experiments. All statistical analyses were performed with SPSS 23.0 (SPSS Inc, Chicago, IL, U.S.A.). The Student’s *t* test was performed to statistically compare between two groups and one-way analysis of variance (ANOVA) was performed for difference among multiple groups. The threshold for statistical significance was set at ^*^P<0.05, ^**^P<0.01.

## Results

### Cell apoptosis was increased and autophagy activation was decreased in foot tendon tissues of DFU rats

**Figure 1:**
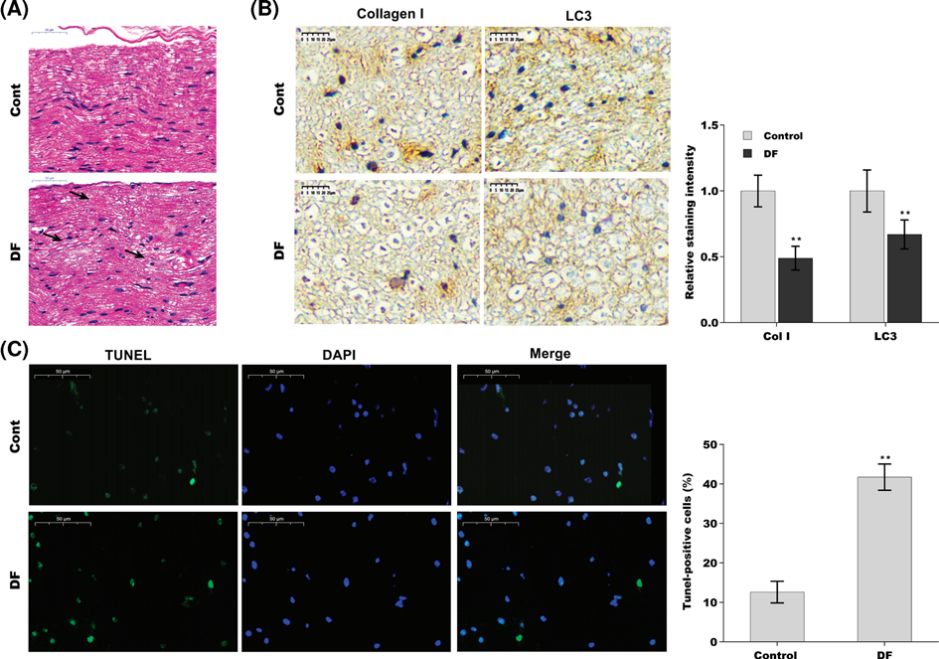
Cell apoptosis was increased and autophagy activation was decreased in foot tendons tissues of DFU rats (**A**) HE staining in foot tendons tissues of DFU rats (*n*=5) and control rats (*n*=5) (×50 μm). (**B**) IHC of the expression of LC3 and Collagen I in foot tendon tissues of DFU rats (*n*=5) and control rats (*n*=5) (×50 μm). (**C**) The cell apoptosis was observed by TUNEL staining in foot tendon tissues of DFU rats (*n*=5) and control rats (*n*=5). The images of TUNEL positive cells were captured by a fluorescence microscope (×50 μm). Abbreviation: HE, Hematoxylin–Eosin.**P<0.01.

Cell apoptosis and autophagy activation were first assessed in foot tendon tissues of DFU rats. To this end, the rat models of DFU were established, and cell apoptosis and autophagy activation were evaluated using HE staining, immunohistochemical (IHC) analysis, and TUNEL assay, respectively. As shown in Figure 1A, collagen bundles were unevenly and sparsely arranged, and showed the minor tear in foot tendon tissues of DFU rats. Adversely, collagen bundles were thick, scathe, and arranged densely in normal tissues. The results from IHC analysis showed that the protein expression of LC3 (the autophagy marker) and Collagen I were decreased in foot tendon tissues of DFU rats compared with controls (Figure 1B). Additionally, compared with normal tissues, the data of TUNEL assay presented that cell apoptosis was significantly increased in foot tendons tissues of DFU rats compared with controls (Figure 1C).

### HG promoted tendon fibroblasts apoptosis

**Figure 2:**
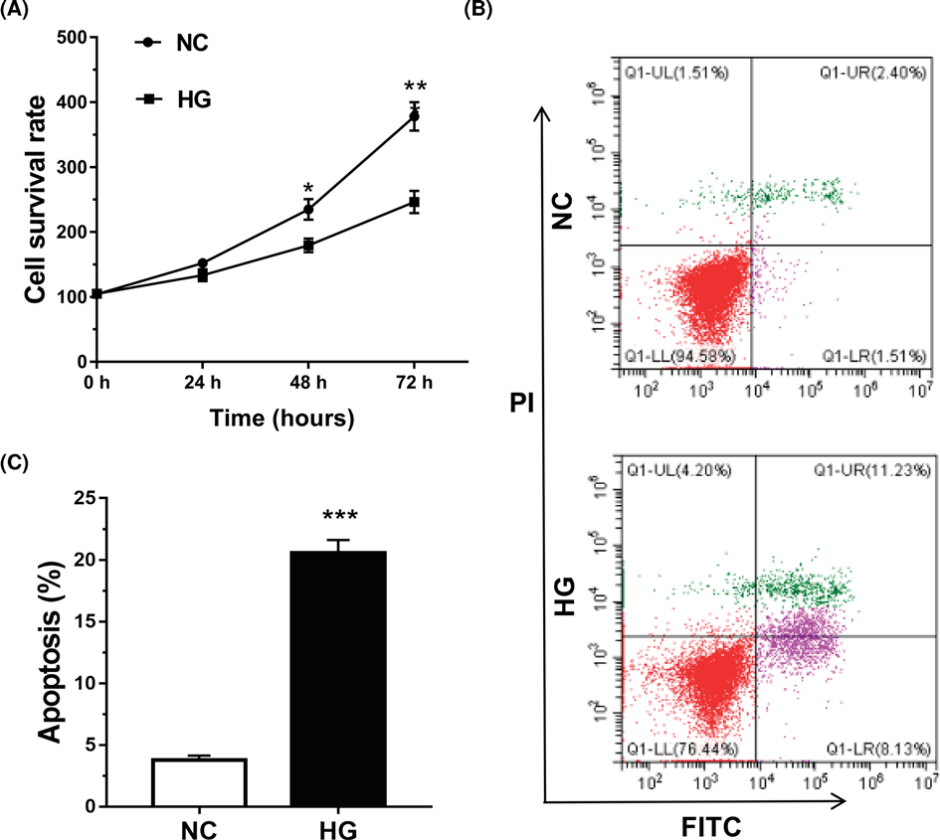
HG promoted tendon fibroblasts apoptosis (**A**) Tendon fibroblast viability was determined using a CCK-8 assay after HG (25 mM) treatment for different times (0, 24, 48, or 72 h). (**B**,**C**) Tendon fibroblast apoptosis was detected using the Annexin V-FITC/PI kit by flow cytometry after HG (25 mM) treatment for 72 h. *P<0.05, **P<0.01, ***P<0.001.

Given the important role of tendon fibroblasts in DFU, we next investigated whether HG induced tendon fibroblasts apoptosis. The results from CCK-8 assay revealed that tendon fibroblasts viability was reduced by HG treatment (Figure 2A). Moreover, the data from flow cytometry analysis revealed that HG promoted the apoptosis of tendon fibroblasts (Figure 2B,C).

### HG repressed autophagy activation of tendon fibroblasts by regulating mTOR activity

**Figure 3:**
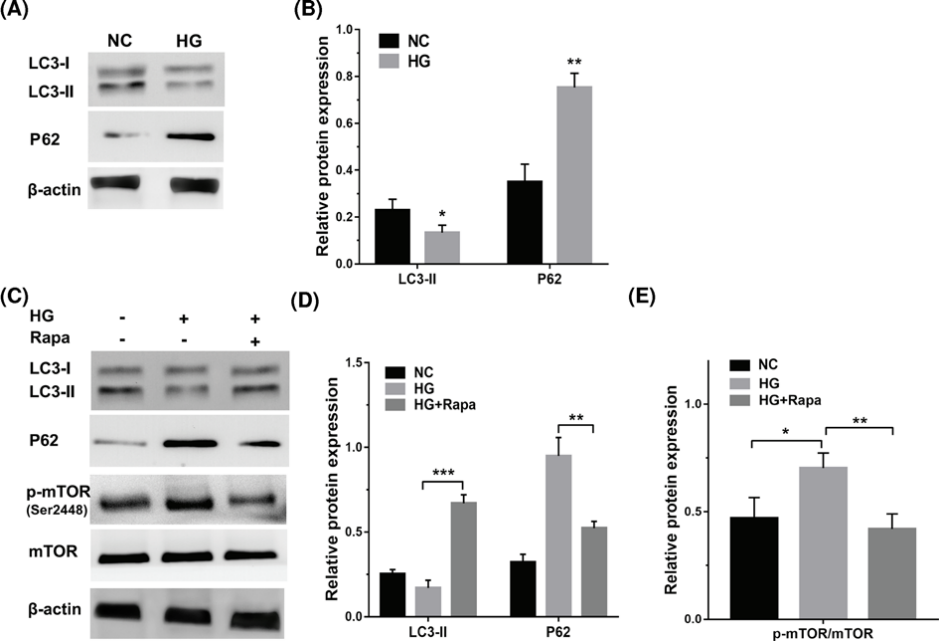
HG repressed autophagy activation of tendon fibroblasts by regulating mTOR activity (**A**,**B**) Western blot analysis for the protein levels of LC3 and p62 in tendon fibroblasts with HG (25 mM) treatment. (**C–E**) Western blot analysis for the protein levels of LC3, P62, p-mTOR, and mTOR in tendon fibroblasts treated with HG (25 mM) in the presence or absence of Rapa (100 nM). *P<0.05, **P<0.01, ***P<0.001.

Previous studies have demonstrated that autophagy exerts an important role in regulating keratinocyte autophagy and subsequent migration [17]. To investigate the role of HG in autophagy activation of tendon fibroblasts, tendon fibroblasts were treated with HG and autophagy activation was assessed using Western blot analysis. Figure 3A,B showed that HG treatment repressed LC3-II protein levels and increased P62 protein levels in tendon fibroblasts, indicating that HG repressed autophagy activation of tendon fibroblasts. The mTOR signal pathway is a crucial regulator of autophagy activity [18]. The activation of mTOR was evaluated in HG-treated tendon fibroblasts through Western blot analysis of phosphorylated mTOR (p-mTOR, Ser^2448^) level. Figure 3C,E showed that HG treatment increased p-mTOR level, suggesting that HG might repress tendon fibroblasts autophagy by regulating mTOR activity. As expected, autophagy activator rapamycin reversed the effect, as evidenced by increased LC3-II level, and decreased p62 and p-mTOR levels (Figure 3C–E).

### Rapamycin alleviated HG-induced injury of tendon fibroblasts

**Figure 4:**
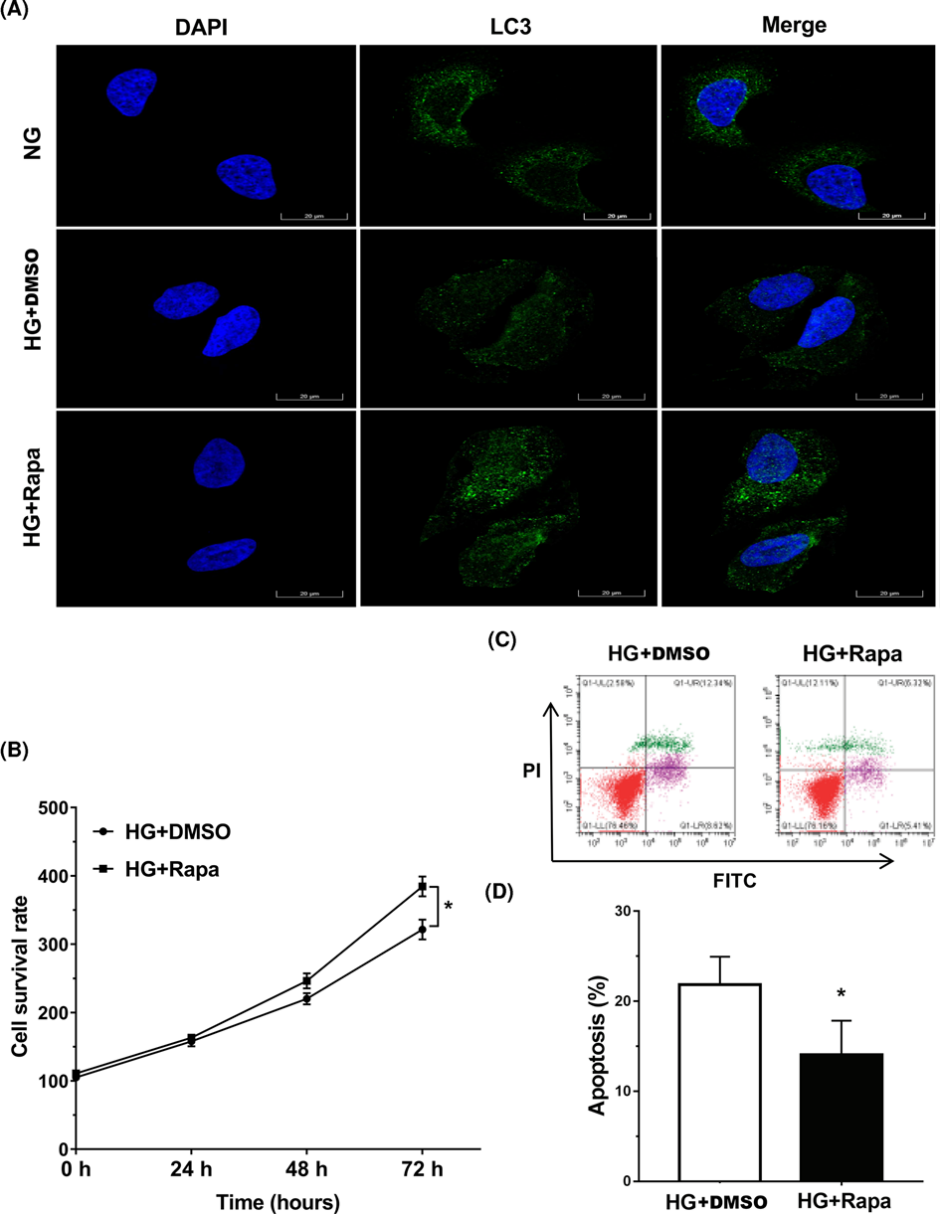
Rapamycin alleviated HG-induced injury of tendon fibroblasts (**A**) The LC3 expression was detected by IF in tendon fibroblasts treated with HG (25 mM) in the presence or absence of Rapa (100 nM). DMSO was used as a vehicle control. (**B**) Tendon fibroblast viability was determined using a CCK-8 assay after HG (25 mM) in the presence or absence of Rapa (100 nM) treatment for different times (0, 24, 48, and 72 h). (**C**,**D**) Tendon fibroblast apoptosis was detected using the Annexin V-FITC/PI kit by flow cytometry after HG (25 mM) in the presence or absence of Rapa (100 nM) treatment for 72 h. *P<0.05.

Finally, we investigated the role of rapamycin-activated autophagy in HG-induced tendon fibroblasts injury. As shown in Figure 4A, rapamycin treatment enhanced autophagy activation in HG-treated tendon fibroblasts, as suggested by increased LC3 green puncta. Functionally, rapamycin treatment increased the viability of tendon fibroblasts in HG model (Figure 4B). Moreover, the data from flow cytometry indicated that rapamycin repressed the role of HG in accelerating tendon fibroblasts apoptosis (Figure 4C,D). These data demonstrate that HG induces tendon injury by repressing tendon fibroblasts autophagy, and rapamycin is helpful to alleviate HG-induced injury of tendon fibroblasts.

## Discussion

Epidemiological investigations show that approximately 60% of diabetic patients will eventually develop DFU [4]. The causes of DFU are multifactorial including inadequate glycemic control, foot deformities, calluses, poor foot care, and dry skin [5]. Tendon fibroblasts play a significant physiological role in remodeling phases of wound healing, and fibroblasts-derived collagen deposition exerts as a basis of the extracellular matrix formation [10,19]. In the present study, we demonstrated that: (i) cell apoptosis is increased and autophagy activation is decreased in foot tendons tissues of DFU rats, (ii) HG accelerates tendon fibroblasts apoptosis *in vitro*, (iii) HG represses autophagy activation of tendon fibroblasts by regulating mTOR activity, (iv) Rapamycin partially alleviates HG-induced injury of tendon fibroblasts. These results verify the important role of tendon fibroblasts autophagy in tendon injury and might provide a new opportunity to treat DFU.

As connective tissue, tendons connect muscles and bones, transfer loads between muscles and bones through complex compositions and a hierarchy is composed mainly of collagen type I, and play an important role in the sports system [20]. Emerging studies have shown that diabetes has complex effects on tendons and bones in a number of ways, including clinical manifestations (pain, stiffness, decreased range of motion), imaging abnormalities, and decreased biomechanical properties (structural and functional changes) [21]. The process of diabetic tendon injury is complex and can involve abnormal immune response, angiogenesis, accumulation of advanced glycation end products (AGEs), and other extracellular matrix changes [22–25]. Currently, the main hypothesis of diabetic tendinopathy is the excessive accumulation of AGEs, abnormal inflammatory response, and insensitivity of new angiogenesis and neuropathy [26]. Currently, the role of tendon fibroblasts in DFU remains unclear. In the present study, we demonstrated that cell apoptosis is increased significantly in foot tendons tissues of DFU rats compared with normal tissues. Collagen bundles are unevenly and sparsely arranged, and showed the minor tear in foot tendons tissues of DFU rats. In cultured tendon fibroblasts, HG treatment represses cell viability and accelerates cell apoptosis. Autophagy activation is decreased in DFU rats and in HG-treated tendon fibroblasts. However, local injection of rapamycin into the defect region could not promote tendon repair in diabetic rats. The route of rapamycin administration and the dose might influence the effect of rapamycin on facilitating tendon repair *in vivo*. It is also necessary to detect the role of rapamycin in tendon repair at different time points.

As an effective mechanism to maintain homeostasis and cellular survival, the role of autophagy in wound healing is gradually being revealed. Li et al., demonstrated that HG-treated keratinocytes exhibits a decreased activation of autophagy compared with control [17]. Inhibition of p38/MAPK signaling or autophagy represses keratinocyte migration in the presence of HG, whereas p38/MAPK activation rescues keratinocyte migration in an autophagy-dependent manner [17]. Autophagy activation is also decreased significantly in diabetic wound epidermis. These results suggest that HG-induced inactivation of autophagy prevents wound healing by repressing keratinocyte migration. On the contrary, the study from Zeng et al., showed that activating autophagy in skin fibroblasts prevents wound healing by repressing collagen synthesis or accelerating collagen degradation [10]. Gao et al., also explored the correlation of HG with autophagy in glomerular mesangial cells (GMCs). Their results revealed that HG increases the activation of GMCs autophagy in a short time (0–12 h), whereas HG represses GMCs autophagy activation during long period (12–72 h) [27].

## Conclusion

The effects of HG on autophagy activation and the roles of autophagy activation in wound healing remain unclear. In the present study, we demonstrated that the activation of autophagy is decreased in foot tendon tissues of DFU rats and in HG-treated tendon fibroblasts. More important, rapamycin effectively alleviates the suppressive role of HG in tendon fibroblasts viability by regulating autophagy.

## Data Availability

The datasets used and/or analyzed during the current study are available from the corresponding authors on reasonable request.

## Abbreviations

AGE: advanced glycation end product
CCK-8: cell counting kit-8
DFU: diabetic foot ulcer
DM: diabetes mellitus
FITC: fluorescein isothiocyanate
GMC: glomerular mesangial cell
HE: Hematoxylin–Eosin
HG: high glucose
IHC: immunohistochemistry/immunohistochemical
p-mTOR: phosphorylated mTOR
TUNEL: TdT-mediated dUTP nick-end labeling
